# Comparative Study of Myocutaneous Latissimus Dorsi Flap Breast Reconstruction With Implant Versus Lipofilling: Complication Rates and Risk Factors

**DOI:** 10.1002/wjs.70494

**Published:** 2026-08-04

**Authors:** Aline Prado de Almeida, Vilmar Marques de Oliveira, Regis Rezende Paulinelli, Fabio Bagnoli, José Francisco Rinaldi, Fabricio Palermo Brenelli, Ivo Carelli Filho, Tainara Rodrigues Miranda

**Affiliations:** ^1^ Department of Obstetrics and Gynecology Irmandade da Santa Casa de Misericórdia de São Paulo São Paulo Brazil; ^2^ Faculty of Medical Sciences of Santa Casa de São Paulo São Paulo Brazil; ^3^ Department of Mastology Associação de Combate ao Câncer de Goiás Goiânia Brazil; ^4^ Department Breast Oncology Universidade de Campinas Campinas Brazil; ^5^ Department of Mastology Faculdade de Medicina do ABC Santo André Brazil

**Keywords:** autologous fat graft, breast cancer, breast implants, latissimus dorsi flap, mammoplasty, surgical complications

## Abstract

**Background:**

Breast cancer is the most prevalent malignancy among women globally, and mastectomy often forms a critical part of its treatment. Breast reconstruction is an important aspect of post‐mastectomy care, contributing to improved psychological well‐being and quality of life. This study compares the outcomes of two distinct Latissimus Dorsi (LD) flap reconstruction techniques: one utilizing implant augmentation (LD‐I) and the other employing autologous fat graft (lipofilling) for volume restoration (LD‐L).

**Methods:**

A retrospective cohort study was conducted, analyzing 116 patients who underwent breast reconstruction with either the LD‐I or LD‐L technique between 2013 and May 2024. Data was collected from two centers in Brazil. The primary outcome was the rate of major complications, defined as those requiring surgical re‐intervention or hospital readmission. Secondary outcomes included the incidence of early (≤ 6 months) and late (> 6 months) complications and the correlation of clinical characteristics in regards to the complications, such as comorbidities and vices.

**Results:**

The study population comprised 50 patients in the LD‐I group and 66 patients in the LD‐L group. The mean age of patients was approximately 50 years in both groups. There were no significant differences between the groups regarding oncologic characteristics, including tumor stage, histological subtype, and receipt of neoadjuvant or adjuvant therapies. While the overall rate of major complications was not statistically different between the two groups (18% LD‐I vs. 7.6% LD‐L, *p* = 0.079), specific complications varied. The LD‐I group exhibited a significantly higher rate of early infections (20% vs. 6.1%, *p* = 0.023) and a greater incidence of late reconstruction loss due to implant‐related issues (10% vs. 0%, *p* = 0.013).

**Conclusion:**

Both LD‐I and LD‐L techniques provide viable options for breast reconstruction. However, the LD‐L technique demonstrated a lower risk of infection and reconstruction loss in this cohort. These findings suggest that LD‐L may offer a favorable alternative for minimizing specific complications and improving long‐term outcomes in post‐mastectomy breast reconstruction. However, these findings should be analyzed with caution and further prospective, randomized studies with larger sample sizes are warranted to confirm these observations and to identify specific patient subgroups that may benefit most from each technique.

## Graphical abstract

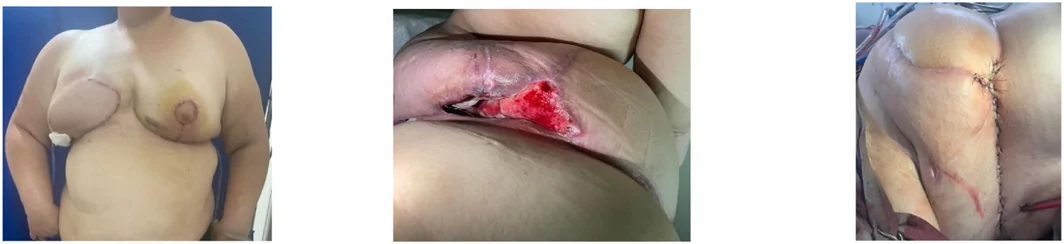

## 1 Introduction

Breast cancer (BC) is the most incident malignant tumor globally (excluding non‐melanoma skin cancer), representing a significant public health issue [1]. In Brazil, an estimated 73,000 new cases are expected between 2023 and 2025, with a risk of 66 new cases per 100,000 women [2]. While surgical treatment is integral to managing BC, procedures like mastectomy can negatively impact a patient's self‐image and well‐being [3]. Breast reconstruction, whether partial or total, offers a means to achieve a favorable aesthetic outcome without compromising oncological treatment. In Brazil, breast reconstruction is a patient's right, though access challenges persist, particularly within the public health system (SUS) [4].

The Latissimus Dorsi Myocutaneous (LDM) flap is a versatile and well‐established technique for breast reconstruction, first described by Iginio Tansini in 1906 and popularized for breast reconstruction in the late 1970s [5, 6, 7]. It can be used with an implant to achieve adequate volume, particularly in medium to large‐breasted patients, or with techniques like the extended LDM flap to avoid implants. However, implant‐based reconstructions carry risks such as malposition, capsular contracture, rupture, and implant loss [8, 9].

Autologous fat grafting, or lipofilling, first reported by Neuber in 1893 [10], involves harvesting fat via liposuction and reinjecting it into the target area. Its use in cosmetic breast augmentation was described in the 1980s [11, 12, 13]. Lipofilling has been shown to be oncologically safe for breast reconstruction patients after BC [14, 15, 16]. In 2014, Santanelli and colleagues made the first report on the use of autologous fat grafting after reconstruction with LDM, with an average volume of 100 mL of fat used and low complication rates [17]. Following this report, in 2019, Brondi et al. described a technique of immediate lipofilling of the LDM flap, potentially avoiding silicone implants and their associated complications. This technique aims for durable results, good symmetry, low cost, and low complication rates [18]. Potential complications of lipofilling include steatonecrosis, oil cysts, infection, and calcifications [19]. Additionally, there's a limit to the volume that can be injected, and some fat reabsorption occurs, sometimes, necessitating further procedures [20].

While both LDM flap reconstruction with implant (LDM‐I) and LDM flap with lipofilling (LDM‐L) are well established, comparative studies are still emerging. This study aims to compare the rates of major complications between these two techniques and investigate risk factors for complications.

## 2 Methods

### 2.1 Study Design and Casuistry

A retrospective observational cohort study was conducted by analyzing 116 medical records of patients with BC who underwent surgical treatment and breast reconstruction using either the LDM‐I or LDM‐L technique. The study period was from 2013 to May 2024. Data were collected from the Mastology outpatient clinics of the Irmandade da Santa Casa de Misericórdia de São Paulo (ISCMSP) and the Associação de Combate ao Cancer de Goiás (ACCG).

### 2.2 Inclusion Criteria

Female patients diagnosed with BC who underwent mastectomy and breast reconstruction (both immediate and delayed) with either the LDM flap with implant or LDM flap with lipofilling were included.

### 2.3 Exclusion Criteria

Patients with inadequate medical records were excluded.

### 2.4 Data Evaluated

The following data were collected from patient records:

Patient‐related: Age at surgery, body mass index (BMI), comorbidities, addictions (e.g., smoking).

Disease‐related: Initial clinical stage, histological and molecular type, neoadjuvant and/or adjuvant therapy, side of the BC.

Reconstruction‐related: Type of reconstruction, implant volume or lipofilling volume, and the service where the procedure was performed.

### 2.5 Outcomes

Were classified as follows:

- Early Complications (manifesting up to 6 months post‐surgery): Infection, hematoma, wound dehiscence, partial or total flap necrosis, seroma, implant loss, reconstruction loss, steatonecrosis
- Late Complications (occurring after 6 months post‐surgery): Implant loss, reconstruction loss, infection, implant capsular contracture (Baker III or IV), implant rupture, loss of lipofilling volume, steatonecrosis.
- Major Complications: Complications requiring hospital admission and/or surgical re‐intervention, regardless of the postoperative time.
- Minor Complications: Complications that did not required surgery or readmission.

Data collected from medical records were entered into a REDCap platform form, elaborated by the researchers. A significance level (alpha) of 5% was used, and statistical analysis was conducted with SPSS version 25.0. The analysis of the association between qualitative variables was performed using the Chi‐square test or Fisher's exact test. Comparisons of quantitative variables were conducted using the Mann–Whitney test or Student's t test, depending on data distribution. The significance level (alpha) was set at 5%. Multivariable analysis performed using logistic regression.

## 3 Results

A total of 116 patient records were reviewed: 54 from ISCMSP and 62 from ACCG. Concerning the type of reconstruction, 50 patients underwent LDM‐I reconstruction, and 66 underwent LDM‐L reconstruction. The groups were homogeneous regarding oncological characteristics, quantitative data, comorbidities, and addictions except for the volume used in the reconstructions, where the implant‐based group showed a significantly higher volume than the fat grafting group (*p* 0.000) (Table 1).

**TABLE 1:** Baseline characteristics and oncological data.

Characteristics	LDM‐I (*n* = 50)	LDM‐L (*n* = 66)	*p* ^a^
QUANTITATIVE DATA—Median (Interquartille range)			
Age (years)	48 (17)	51 (16)	0.474
Body Mass index (N_LDM‐I_ = 43 N_LDM‐L_ = 61)	26.2 (3.8)	26 (6.3)	0.297
Reconstruction volume (mL)	280 (155)	200 (20)	0.000
Follow‐up time (months)	30 (38)	20.5 (15)	0.007
QUALITATIVE DATA—absolute frequency (%)			
Breast side			^**^
Right	25 (51)	36 (54.5)	
Left	23 (46.9)	28 (42.4)	
Bilateral	1 (2)	2 (3)	
Comorbiditie/vices			
Sistemic arterial hypertension (SAH)	10 (20)	19 (28.8)	0.194
Type 2 diabetes Mellitus	5 (10)	7 (10.6)	0.584
Dyslipidemia	1 (2)	4 (6.1)	0.280
Smoking			^**^
Non smoker	32 (65.3)	44 (67.7)	
Current smoker	16 (32)	12 (18.5)	
Former smoker	1 (2)	9 (13.8)	
Histological type *n* (%)			^**^
Invasive ductal carcinoma	43 (86)	54 (81.8)	
Lobular carcinoma	3 (6)	6 (9.1)	
Others	4 (8)	6 (9.1)	
Molecular type *n* (%)			^**^
Luminal A	12 (25.5)	10 (15.6)	
Luminal B	17 (36.2)	32 (50)	
Her 2 enriched	3 (6.4)	6 (9.4)	
Triple negative	7 (14.9)	8 (12.5)	
Luminal hybrid	8 (17)	8 (12.5)	
Clinical staging *n* (%)			
T Tx	1 (2.2)	0 (0.0)	^**^
Tis	1 (2.2)	1 (1.6)	
T1	6 (13.3)	5 (8.2)	
T2	19 (42.2)	19 (31.1)	
T3	11 (24.4)	19 (31.1)	
T4	7 (14.9)	17 (27.9)	
N NO	21 (46.7)	29 (47.5)	^**^
N1	19 (42.2)	21 (34.4)	
N2	5 (11.1)	9 (14.8)	
N3	0 (0.0)	2 (3.3)	
M MX	2 (4.3)	9 (14.1)	^**^
M0	45 (95.7)	54 (84.4)	
M1	0 (0.0)	1 (1.6)	
Neoadjuvant therapy			^**^
No	23 (46)	28 (42.4)	
Chemotherapy	27 (54)	37 (56.1)	
Radiotherapy	0 (0.0)	1 (1.5)	
Adjuvant therapy			
Chemotherapy	22 (44.9)	30 (45.5)	0.552
Radiotherapy	26 (53.1)	51 (77.3)	0.006
Hormone therapy	34 (69.4)	50 (75.8)	0.291

Abbreviations: LDM‐I, latissimus dorsi myocutaneous flap and implant; LDM‐L, latissimus dorsi myocutaneous flap and lipofilling.

^a^ Variable quantitative: Non parametric test Mann‐Whitney; Variable qualitative: Fisher's Exact test.

^**^ *p*‐value not reported due to expected cell counts below 5.

Major complications, requiring reoperation or readmission, occurred in 14 patients overall. This was more frequent in the LDM‐I group (18%) compared to the LDM‐L group (7.6%), though this difference was not statistically significant (*p* = 0.079) (Table 2).

**TABLE 2:** Major complications.

	LDM‐I (*n* = 50)	LDM‐L (*n* = 66)	*p* ^a^
Overall number (%)			0.079
Yes	9 (18)	5 (7.6)	
No	41 (82)	61 (92.4)	

Abbreviations: LDM‐I, latissimus dorsi myocutaneous flap and implant; LDM‐L, latissimus dorsi myocutaneous flap and lipofilling.

^a^ Fisher's Exact Test.

**FIGURE 1:**
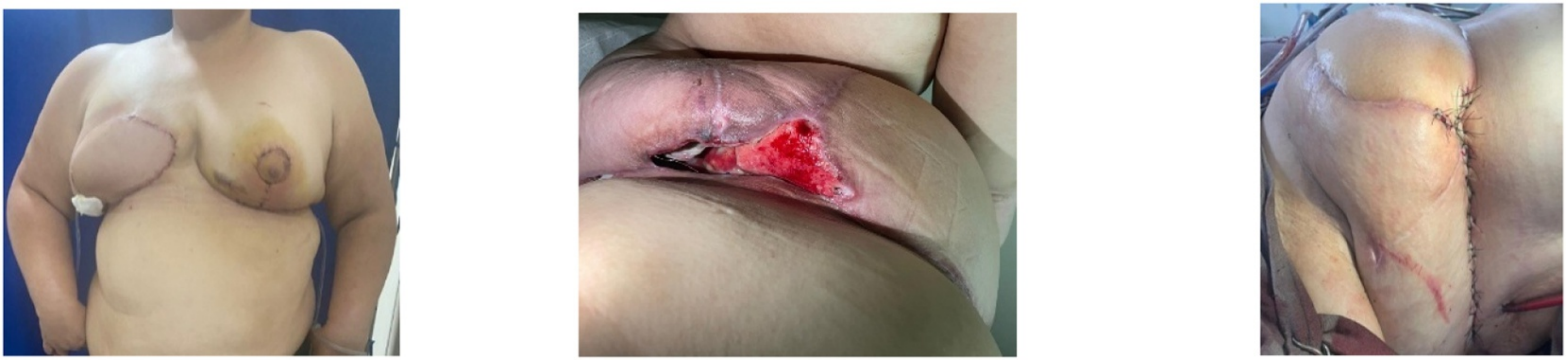
Patient with major complication on the 20th day post LDM‐L reconstruction with surgical wound resuture in the operating room.

The major complications were cases of implant loss, wound resutures (with or without debridement), seroma drainages, and fistulectomies (Figure 1).

**FIGURE 2:**
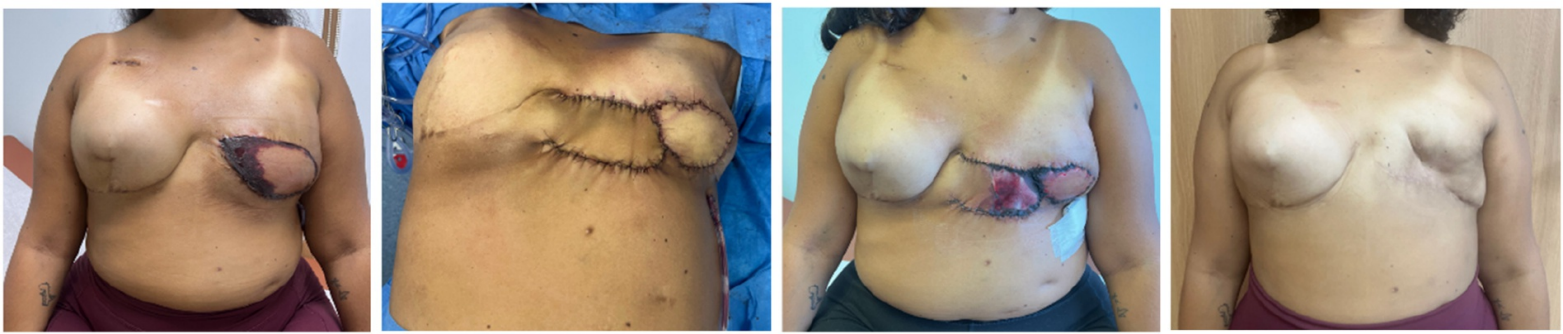
Patient with LDM‐I developed early partial flap necrosis, an attempt at conservative resection of the necrosis was unsuccessful, and removal of the breast implant was necessary.

Early complications occurred in 54 patients (46.55%), including seroma, infection, dehiscence, partial flap necrosis, hematoma, implant loss, and reconstruction loss (Figure 2). There were 27 cases in each group. One reconstruction loss occurred in the LDM‐I group versus none in the LDM‐L group (*p* = 0.431). Infection was significantly more common in the LDM‐I group (10 cases, 20%) compared to the LDM‐L group (4 cases, 6.1%) (*p* = 0.023) (Table 3).

**TABLE 3:** Early complications (≤ 6 months).

Complication	LDM‐I (*n* = 50)	LDM‐L (*n* = 66)	*p* ^a^	*p* ^b^
Total *n* (%)	27 (54)	27 (40.9)	0.162	0.113
Infection	10 (20)	4 (6.1)	0.022	0.023
Dehiscence	6 (12)	8 (12.1)	0.984	0.609
Hematoma	1 (2)	3 (4.5)	0.457	0.420
Seroma	16 (32)	13 (19.7)	0.130	0.097
Partial flap necrosis	3 (6)	1 (1.5)	0.190	0.213
Implant loss^c^	1 (1.5)	0 (0.0)		
Reconstruction loss	1 (1.5)	0 (0.0)	0.249	0.431

Abbreviations: LDM‐I, latissimus dorsi myocutaneous flap and implant; LDM‐L, latissimus dorsi myocutaneous flap and lipofilling.

^a^ Chi‐square test.

^b^ Fisher's exact test; *n* = absolute frequency; % = percentage.

^c^ Variable not suitable for comparison between samples.

Late complications were reported in 13 patients (11.2%), including loss of lipo‐grafted volume, implant loss, reconstruction loss, steatonecrosis, and fistula. Six occurred in the LDM‐I group and 7 in the LDM‐L group. The patients reconstructed with LDM‐L had 2 cases of loss of lipofilling volume, and 4 cases of steatonecrosis (*p* = 0.101). In the implant‐based Group 5 patients lost their reconstruction due to implant loss (*p* = 0.013), which was statistically significant (Table 4).

**TABLE 4:** Late complications (> 6 months).

Complication	LDM‐I (*n* = 50)	LDM‐L (*n* = 66)	*p* ^a^	*p* ^b^
Total *n* (%)	6 (12)	7 (10.6)	0.814	0.520
Implant loss^c^	5 (10)	0 (0.0)		
Loss of lipo‐grafted volume^c^	0 (0.0)	2 (3)		
Steatonecrosis	0 (0.0)	4 (6.1)	0.076	0.101
Fistula	2 (4)	1 (1.5)	0.404	0.396
Reconstruction loss	5 (10)	0 (0.0)	0.009	0.013

Abbreviations: LDM‐I, latissimus dorsi myocutaneous flap and implant; LDM‐L, latissimus dorsi myocutaneous flap and lipofilling.

^a^ Chi‐square test.

^b^ Fisher's exact test; *n* = absolute frequency; % = percentage.

^c^ 1‐ Variable not suitable for comparison between samples.

When comparing the 2 centers involved in this study, in the ISCMSP center, more that 90% of the reconstructions were made using autologous fat graft while in the ACCG center, more that 70% of the surgeries were implant based. Despite the absence of difference in most of the complications rates between the 2 places, the center that did more LDM‐I had an early infection rate almost three times bigger (3,7% at ISCMSP vs. 18,5% at ACCG, *p* = 0.01); in the ISCMSP, where de lipofilling was the major technique, there was a greater find of steatonecrosis (7,5% ISCMSP vs. 0% ACCG, *p* = 0.03).

Adjuvant radiotherapy (RDT‐a) is an important topic when discussing breast reconstruction, especially when it involves breast implants. 78 of 116 patients received RDT‐a (26 in LDM‐I, 52 in LDM‐L; 50% and 79% respectively). Most of the early complications (infection, dehiscence, seroma, hematoma, partial flap necrosis) occurred before RDT‐a. Of the two early implant losses, one occurred after RDT‐a. Of the 13 late complications, 6 patients had received RDT‐a: two of five who lost implants/reconstruction in LDM‐I group, one with lipo‐graft volume loss, and three with steatonecrosis in LDM‐L group. 50% of patients with major complications had undergone RDT‐a including cases of surgical drainage of seroma, re‐suturing of the surgical wound, debridement of partial necrosis of the flap with exposure of the implant and loss of the implant due to a local infection.

## 4 Discussion

Breast reconstruction is a crucial component of breast cancer treatment, aiming for satisfactory oncological and aesthetic outcomes. While oncologically safe, reconstruction carries surgical risks that surgeons strive to minimize through technique selection and patient individualization. The LDM flap is a reliable option, with the main challenge being adequate volume restoration.

Literature reports varied complication rates for LDM flap reconstructions, influenced by surgeon experience, patient selection, and surgical technique. One study on LDM‐I reported seroma (26%) as most prevalent complication, with major complications in 9.1% [21]. Another, with 188 patients, found 58% seroma, 13% infection, and 6.5% early implant loss in LDM reconstructions (66% with implants) [22]. A large database review of 1079 patient and LDM‐I found 5.7% reoperations, 3.3% skin infections, and 1.3% flap necrosis [23]. These findings are generally consistent with the findings in the LDM‐I group in our study.

Lipofilling has become a valuable tool in breast reconstruction, from refinements to total autologous reconstruction. While low body mass index could be a limitation, studies suggest LDM‐L is viable even in underweight patients, though more lipofilling sessions might be needed [24]. Fat reabsorption rates are reported to be around 25%–40% [25] and are an important factor to be considered when planning on using autologous fat graft in the reconstruction technique; however, in our cohort of 67 LDM‐L patients, only two experienced significant volume loss requiring further sessions (2,98%).

Data on LDM‐L is more recent [26]. A systematic review found total reconstruction loss of 3%, seroma 18.2%, and steatonecrosis 14.5%. Another study on 100 LDM‐L patients reported no major complications and 6.76% minor complications (partial flap necrosis, seroma, steatonecrosis, dorsal wound dehiscence) [27]. Literature supports LDM‐L as oncologically safe with good cosmetic outcomes and manageable complications.

Comparing these techniques helps tailor choices. As this was a retrospective chart‐review study, no criteria were established for selecting the reconstruction technique for each patient; it is believed that this choice was made based on the surgeon's and surgical team's experience and skills at the time of surgery, as well as the available resources.

Our study involved two centers with different predominant techniques. ISCMSP (mostly LDM‐L) had a 1.9% reconstruction loss rate. ACCG (mostly LDM‐I) had nearly three times this rate; this difference can be explained by the fact that in the ACCG center, the use of implant was 15 times more frequent than in ISCMSP. It is important to point that both are teaching centers, with breast surgeons in training participating in the reconstructive surgeries and pos operative care which may influence complication rates. Also, each center has its own surgical protocol, from the patient and type of surgery selection to the way they managed the complications.

All 6 reconstruction losses in our study occurred in the LDM‐I group due to implant loss (early or late). The statistically significant higher reconstruction loss in LDM‐I was in late complications (*p* = 0.01). Major complications were also doubled in the LDM‐I group, though not statistically significant (*p* = 0.08). This aligns with Demiri et al. (2018), who found higher complication and reconstruction loss rates in implant‐based reconstructions (albeit in delayed post‐radiation cases) [28]. The early infection rate was significantly higher in our LDM‐I group (20%) compared to LDM‐L (6.1%), exceeding the ∼3% reported in some literature, possibly due to the teaching hospital setting [23].

Although the LDM‐I group had higher prevalence of smoking, this did not show a statistically significant impact on complication rates in this cohort. Radiotherapy can increase complications with implants. Losken et al. found radiotherapy a significant risk factor for complications in LDM‐I reconstructions (34% complication rate in irradiated patients) [29]. In our sample, most RDT‐a patients were in the LDM‐L group, which had lower overall complication rates and no reconstruction losses. RDT‐a was not a risk factor for reconstruction loss due to implant loss in our LDM‐I group. It is important to emphasize that this was not the primary focus of the study, and that the sample size (*n*) may have influenced this outcome.

Comparative studies of LDM‐I versus LDM‐L are scarce, and techniques for lipofilling vary. Leuzzi et al. (2019) found implant reconstructions had more complications and reoperations, with better patient satisfaction in lipofilled reconstructions due to natural results [30]. Tilkin et al. (2025), in a systematic review of 5143 patients, found heterogeneous techniques and greater follow‐up in implant groups. They reported similar overall complication rates (∼25%) but double the reoperation rate for major complications in LDM‐I (4.9%) versus LDM‐L (2.4%) [31].

Our findings corroborate this literature, showing significantly lower early infection rates and no reconstruction loss in the LDM‐L group. Avoiding alloplastic material is another benefit of LDM‐L. Thus, LDM‐L appears to offer a safer alternative concerning these specific complications.

It is important to point the limitations of this study: the fact that was a retrospective review of charts, with no linear follow up of the patients and their complications. It is crucial to highlight the disparity in reconstruction volumes; specifically, the implant‐based group—which exhibited the highest rate of reconstructive failure—presented significantly higher volumes compared to the lipofilling group. This disparity may represent a significant bias within the study, potentially confounding the analysis of the outcomes.

Also, with 2 different centers, each one with its own protocols of surgical indications and post‐surgical care, with learning surgical residents participating on the reconstruction procedure and clinical management, all of which can be considered study biases. Another important caveat regarding the study's limitations is the fact that many of the findings may be underpowered, from the statistical point of view, as is the case with major complications (*p* 0.08). In regards to the LDM‐L reconstruction, it is important to point that in the studies evaluated, there is substantial heterogeneity in surgical technique and in the volume of fat grafted, which may contribute to differing outcomes. It should also be noted that this group had a shorter follow‐up, which may have underestimated the true complication rate.

To account for the influence of potential confounding factors, such as discrepancies in the follow‐up period and inter‐center variability, a more detailed statistical analysis was employed. This approach aimed to enhance the robustness of the findings regarding the incidence of major complications. In the multivariable analysis for major complications, no variables reached statistical significance.

However, these results should be interpreted with caution, suggesting that prospective studies with predefined and uniform criteria may be of interest.

## 5 Conclusion

Comparing LDM‐I and LDM‐L breast reconstruction techniques, this study found a trend towards fewer major complications in the LDM‐L, although this was not statistically significant (*p* = 0.079). However, specific complications showed significant differences, especially a significantly higher rate of early infection and late reconstruction loss in the LDM‐I group (*p* = 0.023 and *p* = 0.013 respectively). Assessed comorbidities and habits did not show statistical significance concerning observed complications in either group. These findings suggest that LDM reconstruction with lipofilling may present a more favorable profile regarding early infection and long‐term reconstruction survival.

Based on these findings, reconstruction with LDM‐L can be considered a promising alternative within the spectrum of breast reconstruction techniques. This procedure is characterized by low complication rates and eliminates dependency on exogenous materials for surgical success. Given a suitably qualified surgical team and meticulous patient selection, autologous fat grafting democratizes the provision of sufficient latissimus dorsi volume, thereby ensuring that the patient receives both effective oncological treatment and an appropriate aesthetic result. Notably, while LDM‐I remains a well‐established approach for myocutaneous flap breast reconstruction, its indication is expected to become more restrictive given this new surgical option.

While these findings offer valuable insights, important limitations must be acknowledged, including inter‐center variability, institutional bias, limited statistical power, technical heterogeneity, and follow‐up discrepancies. Nonetheless, these observations warrant cautious interpretation, underscoring the necessity for future prospective, randomized studies with larger cohorts to solidify these findings and guide individualized technique selection.

## Author Contributions

**Aline Prado de Almeida:** conceptualization, data curation, formal analysis, investigation, methodology, project administration, resources, software, supervision, visualization, validation, writing – original draft, writing – review and editing. **Vilmar Marques de Oliveira:** conceptualization, methodology, writing – review and editing. **Regis Rezende Paulinelli:** supervision. **Fabio Bagnoli:** formal analysis, validation, writing – review and editing. **José Francisco Rinaldi:** formal analysis, validation, writing – review and editing. **Fabricio Palermo Brenelli:** validation, writing – review and editing. **Ivo Carelli Filho:** validation, writing – review and editing. **Tainara Rodrigues Miranda:** data curation.

## Funding

The authors have nothing to report.

## Disclosure

The authors have nothing to report.

## Ethics Statement

This study was approved by the medical ethics committee. CAAE: 68679623.9.2001.0031.

## Consent

Informed consent was obtained from all individual participants included in the study.

## Conflicts of Interest

The authors declare no conflicts of interest.

## Data Availability

The data that support the findings of this study are available on request from the corresponding author. The data are not publicly available due to privacy or ethical restrictions.
